# Learned distractor rejection: Robust but surprisingly rapid

**DOI:** 10.3758/s13414-025-03051-4

**Published:** 2025-04-11

**Authors:** Isaac Savelson, Christopher Hauck, Mei-Ching Lien, Eric Ruthruff, Andrew B. Leber

**Affiliations:** 1 Department of Psychology, The Ohio State University, 1835 Neil Ave, Columbus, OH 43210 USA; 2 School of Psychological Science, Oregon State University, Corvallis, OR USA; 3 Department of Psychology, The University of New Mexico, Albuquerque, NM USA

**Keywords:** Attentional capture, Visual search, Inhibition

## Abstract

The ability to reduce the distraction associated with repetitive irrelevant stimuli is critical to goal-directed navigation of the visual environment. Research has supported the existence of such an ability, which has often been referred to as *learned distractor rejection* (Vatterott & Vecera *Psychonomic* *Bulletin & Review*, *19*, 871–878, 2012). However, despite being theoretically relevant to many prominent accounts of distractor ignoring, few studies have directly tested learned distractor rejection since its conception. In the current study we present three direct replications of Vatterott and Vecera’s method that were separately conducted by two independent groups of researchers. Using the conventional split-block analysis, all three replications produce nearly identical results that fail to replicate the original study’s finding. However, using analyses on a finer-grained timescale we found compelling evidence for the existence of a learned ignoring of salient distractors. Critically, this learning occurred much more rapidly than has been previously assumed, taking only two or three encounters with the distracting item before efficient rejection emerged.

## Introduction

The ability to process visual information in a goal-directed manner is fundamental to navigating the visual world. One of the greatest challenges our visual system must overcome in this pursuit is avoiding attentional capture by salient-but-irrelevant distractors (e.g., color singletons). For many years, theories of attentional capture assumed that by simply adopting a search strategy focused on the specific features of a target (i.e., *feature search mode*), one can avoid attentional capture by any salient distractor not matching the target template features (Bacon & Egeth, 1994; Burra & Kerzel, 2013; Folk et al., 1992; Lamy & Egeth, 2003; Leber & Egeth, 2006).

This assumption that search strategies—or templates—were entirely dependent on target properties was first challenged by early formulations of distractor-suppression hypotheses (e.g., the *signal suppression hypothesis*; Gaspelin et al., 2015; Sawaki & Luck, 2010). According to these accounts, avoiding attentional capture by salient distractors is not accomplished solely by tuning for target items, but rather also through suppression mechanisms that act specifically on distractors to reduce their ability to garner attentional priority (see also Chang & Egeth, 2019, 2021).

### Learned distractor rejection

Vatterott and Vecera (2012) proposed that, to implement effective distractor rejection, individuals need to first gain experience with specific features of the distractor. To test this hypothesis, they used a feature-search version of the additional singleton paradigm (Theeuwes, 1992; see Bacon & Egeth, 1994, Experiment 3) and modified it such that the color of a singleton distractor changed at the start of each block but then remained consistent throughout that block. The elegance of this manipulation was that it allowed Vatterott and Vecera (2012) to observe participants’ performance at the beginning of each block, before they had gained experience with distractors in that particular color. Using a *split-block analysis,* comparing attentional capture effects in the first and second half of blocks (24 trials each), they found a large attentional capture effect in the first block half, followed by a complete elimination (if not a reversal) in the second block half. Furthermore, this pattern of capture attenuation was remarkably consistent across the four blocks. These results provided evidence for an additional requirement of the implementation of distractor rejection: experience with the specific features of the distracting stimulus (see also Vatterott et al., 2018).

Vatterott and Vecera’s (2012) finding has been highly influential and is frequently cited as evidence of *learned distractor rejection/suppression.* A primary example of this influence can be found in recent versions of the signal suppression hypothesis (Gaspelin et al., 2015; Sawaki & Luck, 2010), which place great emphasis on experience with specific distractor features (Gaspelin & Luck, 2018, 2019; Hauck et al., 2024; Luck et al., 2021). Another example can be found in habituation accounts of distractor rejection, which posit that the automatic orienting of attention to an irrelevant color singleton is subject to habituation with repeated exposure to the stimulus (De Tommaso & Turatto, 2019; see also Turatto, 2023). Importantly, the theoretical weight placed on the necessity of learning to ignore distractors is supported by recent work comparing learned and cued distractor ignoring. Several studies have argued that actively trying to suppress a distractor results in a much weaker form of distractor suppression (Addleman & Störmer, 2022, 2023; Gaspelin et al., 2019; Stilwell & Vecera, 2019; Wang & Theeuwes, 2018a) or even the absence of distractor suppression (Hauck et al., 2024).

Despite the importance of Vatterott and Vecera’s (2012) findings to prevalent models of distractor suppression, we know of no published studies that conducted a direct replication. However, several studies have conceptually replicated Vatterott and Vecera’s study, testing the learned rejection of a salient color singleton without using the original study’s exact design (De Tommaso & Turatto, 2019; Gaspelin & Luck, 2018; Ramgir & Lamy, 2023; Savelson & Leber, 2024.; Vatterott et al., 2018). These studies have produced mixed results, with some failing to replicate the original decline in capture effects across block halves. Only one of these studies (Vatterott et al., 2018), which was from the same lab as the original study, produced a clear replication, with a magnitude roughly similar to the original study. De Tommaso and Turatto (2019) found a significant effect, although the reduction in capture effects from the first half to the second half for each block was only about a quarter of the magnitude of what Vatterott and Vecera (2012) had found. Ramgir and Lamy (2023) obtained an even smaller effect. The remaining conceptual replications were generally unable to even obtain a significant effect. Gaspelin and Luck’s (2018) response time (RT) data showed a trend in the predicted direction, but it was nonsignificant. Savelson and Leber (2024) reported recurrent difficulties obtaining this effect across multiple experiments. Taken together, these results have called the learned distractor rejection effect into question. It is unclear whether this is because the original finding is not replicable or because the conceptual replications included critical changes in methodology.

#### Current study

To resolve this issue, we present three direct replication attempts of Vatterott and Vecera (2012), separately conducted by two groups of researchers. Experiment 1 was conducted by Hauck, Lien, and Ruthruff, who expected to replicate the original findings but intended to test an alternative explanation. Savelson and Leber (Experiments 2 and 3) conducted a direct replication after previously encountering some difficulty when conceptually replicating the learned distractor rejection effect (see Savelson & Leber, 2024). Note that the two groups worked independently, without awareness of the other group’s work. To preserve that independence, we report below the original analyses conducted by each research group. As a result, the following experiments each use slightly different analysis approaches. Nevertheless, both sets of experiments obtained essentially the same results.

To preview, using sample sizes roughly four times larger than the original two experiments reported by Vatterott and Vecera (2012; *N* = 16 for each experiment), both groups produced what initially appeared to be failures to replicate their learned distractor rejection effect. That is, when we analyzed our data on the same timescale as Vatterott and Vecera (i.e., the split block analysis), our results were strikingly different from the original study. However, when we conducted our analyses on a finer-grained timescale we found robust evidence for learned distractor rejection, with a surprisingly rapid time course.

## Experiment 1

We (Hauck, Lien, & Ruthruff) set out to directly replicate the original Vatterott and Vecera (2012) experiment as closely as possible based on the information reported in their Method and Results sections. However, we additionally sought to more precisely estimate the duration of the early-trial capture effect, we conducted moving RT analyses by calculating RT as a function of trial number within blocks (following the running average approach developed by Gaspelin and Luck, 2018, with eye-tracking data in Experiment 4; see their Fig. 8).

### Method

Experiment 1 was not preregistered, but the original data are available online (https://osf.io/yxg8e).

#### Participants

Although the significant interaction between block half (RTs from the first or second half of the block) and singleton condition (present vs. absent) was observed with a sample size of only 16 in Vatterott and Vecera (2012, Experiment 1), we aimed for a sample size of 70. This was in part to match the sample size of a planned follow-up experiment that would include additional independent variables. The present sample size would achieve a power (1 − *β*) of 0.99 (α = .05, based on an estimated effect size of 0.59 from Vatterott & Vecera, 2012; Lakens, 2013).^1^ Seventy-three undergraduates at Oregon State University participated in exchange for course credit. Three participants were removed from all analyses after they failed to meet our inclusion criterion of 80% accuracy. Data from the remaining sample of 70 participants (50 women, 20 men, mean age = 20 years)^2^ were used in the final analyses. All reported having normal or corrected-to-normal acuity and demonstrated normal color vision using the Ishihara Test for color deficiency. The study was approved by the Oregon State University Institutional Review Board committee, and all procedures were in accordance with the 1964 Helsinki declaration and its later amendments or comparable ethical standards. All data were collected in person during the years of 2019–2020.

#### Apparatus and stimuli

**Fig. 1:**
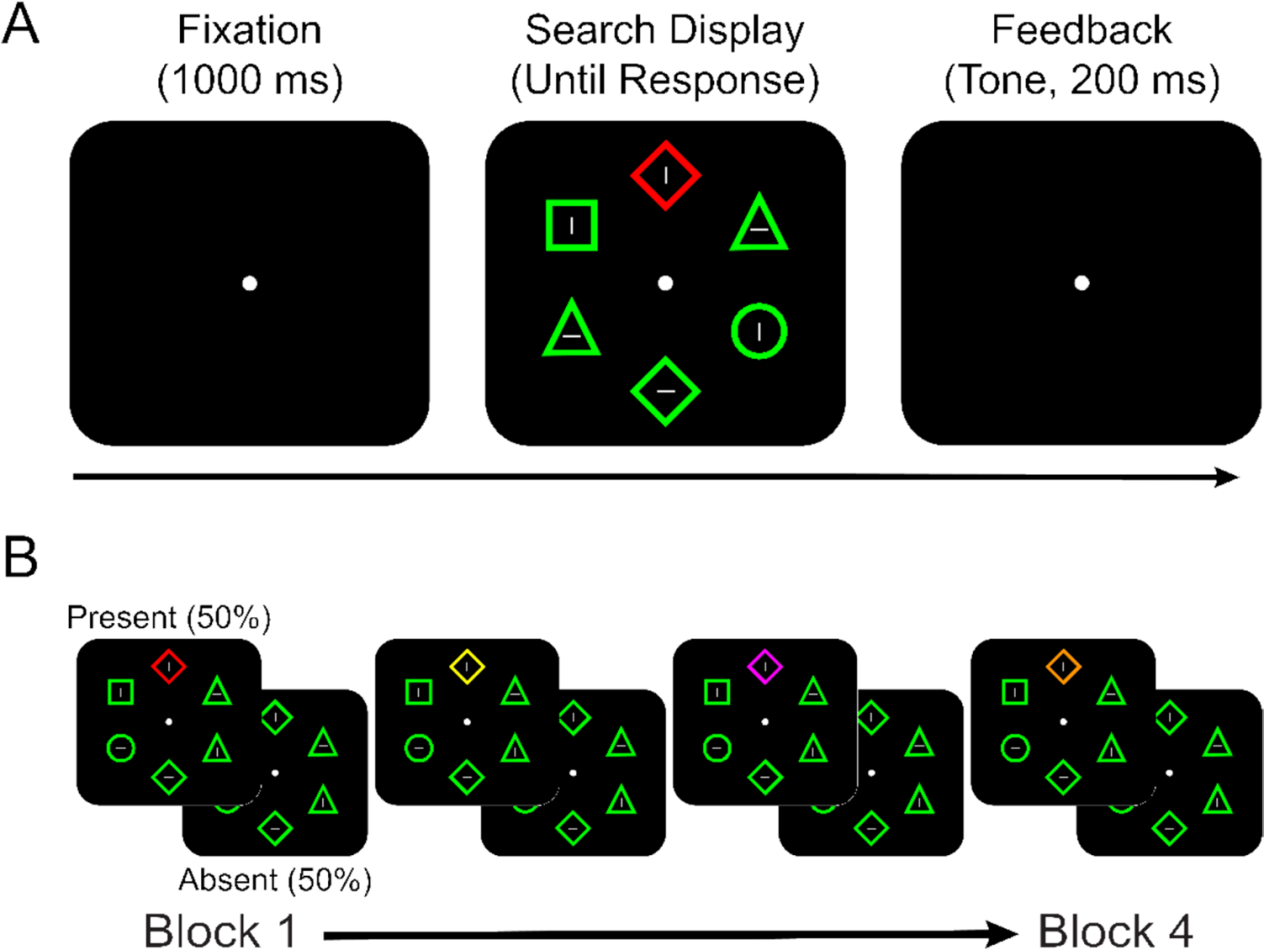
**A** Example trial procedure for a singleton present trial in Experiment 1. For all participants, the target was always a green circle. **A** Distractors colors were consistent within, but varied between, blocks and could appear in red, orange, yellow, or purple. Distractors appeared on half of the trials in each block. (Color figure online)

Stimulus presentation, timing, and data collection were controlled through Windows computers driven by E-Prime 2.0 software (Schneider et al., 2002). The stimuli were presented on a 19-in. monitor. The visual angles given below were based on an average viewing distance from the monitor of about 55 cm. The fixation display consisted of a central white circle (RGB: 255, 255, 255; 0.63° in diameter). The search display consisted of the fixation display and six peripheral shapes arranged in an imaginary circle (see Fig. 1). The shapes included one target circle (2.60° in diameter) and five distractors randomly selected from diamond (3.64° in width and height), triangle (2.91° width × 2.91° height), and square (2.60° in width and height) with the restriction that each shape was used at least once in each trial. Each peripheral shape was equidistant from the central dot (4.68°, center to center) and from adjacent peripheral shapes (4.68°, center to center). Each shape also contained a filled white bar (1.04º in width × 0.52º in height) that was either horizontal or vertical. Each shape was drawn with thin (0.21°) green lines (RGB: 0, 255, 0). When the color singleton distractor was present, one of the distractor shapes was either red (RGB: 255, 0, 0), yellow (RGB: 255, 255, 0), purple (RGB: 255, 0, 255), or orange (RGB: 255, 150, 0). Distractor-color RGB values were taken from Vatterott and Vecera’s (2012) study.

#### Design and procedure

As shown in Fig. 1, each trial began with the presentation of the fixation display for 1,000 ms, followed by the search display for 5,000 ms or until participants responded. The participants’ task was to find the target circle and indicate the orientation (vertical vs. horizontal) of the bar inside by pressing the “Z” key or “M” key, respectively. Auditory tone feedback (a 200-ms error beep) followed incorrect responses, whereas silence (200 ms) followed correct responses. Immediately after the feedback, the next trial began with the fixation display.

Following Vatterott and Vecera (2012), we used green as the relevant (target) color and used the remaining four colors (red, yellow, purple, and orange) as irrelevant (distractor) colors. Participants first performed one 60-trial practice block without the irrelevant color (i.e., all shapes were green; the color singleton absent condition). They then completed four experimental blocks of 48 trials each. The experimental blocks were identical to the practice block, except that one distractor object appeared in an irrelevant color on 50% of the trials (the color singleton present condition). The color of the irrelevant color singleton was fixed within a block but changed between blocks. The assignment of the four irrelevant colors to the four blocks was random, without replacement. The locations of the target shape and the color singleton distractor were randomly determined, with the restriction that they never coincided at the same location.

Participants were instructed to respond to the orientation of the bar inside the target circle as quickly and accurately as possible. Although we did not have a chin rest to hold participant’s head stable, we instructed them to maintain their gaze on the fixation circle throughout the experiment. After each block, mean RT and accuracy for that block were displayed. The next block began when participants pressed the space bar to continue.

### Results and discussion

Data trimming procedures closely followed those of Vatterott and Vecera (2012). We excluded trials from analyses if RTs exceeded three standard deviations from the participant’s overall mean (1.7% of trials). Error trials and trials following errors were also excluded from RT analyses (7.7% of trials). Although Vatterott and Vecera reported an unusually low removal rate of fewer than 2% of trials, more recent investigations using similar paradigms report comparable removal rates to ours (e.g., <2.5% for RT outliers and 5.2% for errors in De Tommaso & Turatto, 2019). Whenever sphericity was violated according to Mauchly’s test of sphericity, *p* values were adjusted using the Greenhouse–Geisser epsilon correction for nonsphericity (Greenhouse & Geisser, 1959). Partial eta squared (*η*_*p*_^2^) was reported as a standardized effect size. For within-subject *t* tests, we reported Cohen’s *d*_z_ for effect size.

#### Response times

##### Split-block analyses

**Fig. 2:**
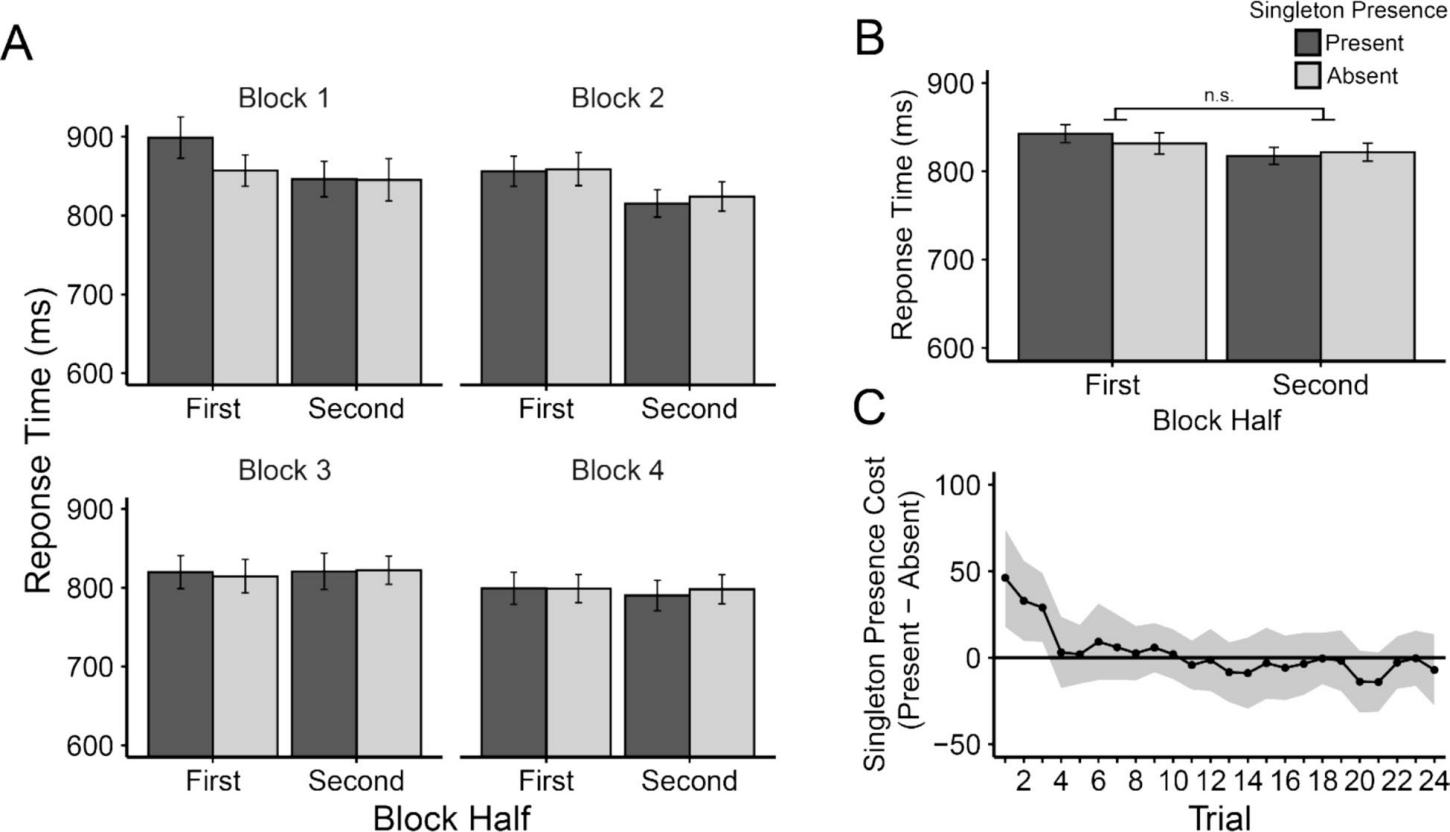
Experiment 1 results. ***A*** Mean response times (in ms) as a function of block half (first vs. second halves of blocks) and singleton presence (present vs. absent) for each block (1–4). ***B*** Response time presented in the same manner as panel A but pooled across blocks. ***C*** Moving RT singleton presence costs (present-absent) across trials within pooled blocks. Moving RTs were computed across five trials (trial *N* − 2 through trial *N* + 2). Error bars represent within-subject 95% confidence intervals (Cousineau, 2005; Morey, 2008)

Mean RTs are depicted in Fig. 2A. We first emulated two of Vatterott and Vecera’s (2012) two-way analyses of variance (ANOVAs) using a larger factorial design. A 2 × 2 × 4 repeated-measures ANOVA was conducted with factors singleton presence (present vs. absent), block half (the first vs. second half of a block), and block (1–4). We found significant main effects of block half, *F*(1, 69) = 7.85, *p* = .007, *η*_*p*_^2^ = .10, and block, *F*(2.252, 155.420) = 17.77, *p*_*adj*_ < .001, *η*_*p*_^2^ = .21, with faster RTs in the second half of a block (819 ms) than the first (837 ms) and faster RTs in later blocks (857 ms, 835 ms, 816 ms, and 792 ms for Blocks 1–4, respectively). The main effect of singleton presence was not significant, *F*(1, 69) = .45, *p* = .504, *η*_*p*_^2^ = .007; mean RT was 827 ms for the singleton present trials and was 824 ms for the singleton absent trials. The two-way interaction between block half and block was significant, *F*(2.587, 178.504) = 3.51, *p*_*adj*_ = .022, *η*_*p*_^2^ = .05. We observed large RT differences between the first and second halves in Block 1 (33 ms) and Block 2 (37 ms), whereas the difference was negligible in Block 3 (−3 ms) and Block 4 (4 ms).

The most critical result was the interaction between block half and singleton presence. Although there was a numerical trend towards larger singleton presence costs (an index of attention capture by the color singleton distractor) in the first halves of the blocks (10 ms) than the second halves of the blocks (−6 ms), *F*(1, 69) = 3.25, *p* = .08, *η*_*p*_^2^ = .05 (see Fig. 2B), this effect was only a small fraction of the effect reported in Vatterott and Vecera’s (2012) Experiment 1 (approximately 77 ms vs. −29 ms for the first and second halves, respectively, as estimated from their Fig. 2).

Singleton presence and block did not significantly interact, *F*(2.731, 178.504) = 1.73, *p*_*adj*_ = .167, *η*_*p*_^2^ = .02. The three-way singleton presence by block half by block interaction was also not significant, *F*(3, 207) = 1.41, *p* = .240, *η*_*p*_^2^ = .02, indicating that the possible attenuation of singleton presence costs across block halves did not vary as a function of block. Nevertheless, further exploratory t-tests on the RT capture effect for each block half and each block revealed that the capture effect reached statistical significance only in the first half of the first block (95% confidence interval [CI]: 40±29 ms), *t*(69) = 2.79, *p* = .007, *d*_*z*_ = 0.33, all other* t*s ≤ 0.94, *p*s ≥ .349.

##### Finer-grained analyses (moving RT)

Our next analysis took a finer-grained approach to characterizing learned distractor rejection, which we term a moving RT analysis (see Gaspelin & Luck, 2018, Experiment 4). We first numbered each trial separately for each singleton condition (present vs. absent) within each block (prior to any trimming of trials). We then excluded trials from analyses using the same RT criteria described above. Next, we calculated a moving average of mean RT across sets of five consecutive trials (i.e., Trial *N* − 2 through trial *N* + 2) within a block, which were then averaged across the four blocks. Despite these data points actually representing averages across multiple trials, we henceforth refer to these windows as *trials* for the sake of simplicity. By analyzing RTs in this manner, we were able to observe decreases in attentional capture on the fine-grained timescale of individual distractor encounters. The results are shown in Fig. 2C.

We performed a set of 24 paired-samples *t* tests to compare RTs on singleton present and absent trials at each data point. Note that we did not correct for familywise error among this large set of tests, and we leave the interpretation of the raw *p* values and effect sizes to the reader. These tests revealed significant singleton presence costs only for the first three encounters with the distractor, *t*s(69) ≥ 2.49, *p*s ≤ .015, *d*_*z*_s ≥ .30, all other *t*s ≤ 1.52, *p*s ≥ .134, *d*_*z*_s ≤ .18.

Singleton presence costs were substantial very early in the block, but these costs rapidly declined after only three encounters with the color singleton distractor. This rapid speed of rejection helps explain our difficulty detecting Vatterott and Vecera’s (2012) learned distractor rejection effect using the first versus second block half analysis. That is, splitting blocks into bins of 24 trials was far too coarse to detect this short-lived early-trial capture effect. In fact, Vatterott and Vecera acknowledged that their split-block analysis might not be optimal, noting that an informal examination of the trial-by-trial RT pattern (the details of which were not reported) found that capture occurred in the first eight to nine encounters/trials with the distractor. Nevertheless, Vatterott and Vecera were able to find large capture effects when using the split-block analysis (24 trials) in the original study. Here, we did not find their split-block learned distractor rejection effect. Instead, we observed a much faster distractor rejection time course than Vatterott and Vecera estimated.

#### Error rates

We conducted the same three-way ANOVA—described for RTs above—on error rates. Only the main effect of block was significant, *F*(2.61, 179.91) = 6.28, *p*_*adj*_ < .001, *η*_p_^2^ = .08, with more errors in the first block (.05) relative to the remaining blocks (min = .03, max = .04). No other significant effects were found for error rates, *F*s ≤ 2.59, *p*s ≥ .112, *η*_p_^2^s ≤ .03.

## Experiment 2

Experiments 2 and 3, by Savelson and Leber, were also direct replication attempts of Vatterott and Vecera (2012) and were conducted independently from Experiment 1. Experiment 2 was conducted using an online platform, whereas Experiment 3 was conducted in person (as was Experiment 1).

### Method

All experimental procedures and statistical analyses were preregistered online (10.17605/OSF.IO/GC6WK).

#### Participants

We recruited 65 participants (29 women, 29 men, two nonbinary, mean age = 29.3 years) through Prolific.co, a recruitment platform for online behavioral experiments. Previous work has found inconsistent effect sizes for the critical learned distractor rejection effect, with power analyses suggesting as few as 32 participants (De Tommaso & Turatto, 2019), and as many as 151 participants (Savelson & Leber, 2024) would be necessary to achieve 80% power. In the absence of a clear effect size, we chose to simply recruit a much larger sample size than that of Vatterott and Vecera (2012). Participants received $5 each for their participation, which took an average of 10 min to complete. All participants reported normal or corrected-to-normal visual acuity and color vision. Eligible participants were restricted to those aged 18–40, were fluent in English, and had at least a 96% approval rating and 50 prior submissions on Prolific.co. This study was determined exempt from IRB review by the Institutional Review Board at The Ohio State University. All data were collected in the summer of 2023.

#### Apparatus and stimuli

All participants completed the experiment online on personal devices. Therefore, we did not have direct control over the visual environment. Stimuli were drawn using HTML Canvas Graphics and presented via jsPsych (Leeuw et al., 2023). Apart from being conducted online, experimental stimuli, design, and procedures were very similar to those used in Experiment 1, with a few minor differences listed below. The visual angle of our stimuli could not be directly determined due to the experiment’s online format. To remain consistent with the in-lab experiment we conducted, we report visual angles assuming a 24-in. monitor with a 1,920 × 1,080 resolution at a viewing distance of 70 cm (see Experiment 3). Our search display consisted of six outline shapes (0.09° line thickness) evenly spaced along the circumference of an imaginary circle centered on fixation (3.71° radius). The target was a shape-defined green circle (2.09° diameter) while distractor shapes were randomly chosen to be squares (1.86° on a side), diamonds (1.76° on a side), and triangles (2.16° base × 2.08° height). The search target appeared equally often at each location throughout the experiment but was pseudorandomized across trials.

#### Design and procedure

Whereas Experiment 1 randomized the presentation order of the distractor colors, Experiment 2 counterbalanced across participants using a 4 × 4 balanced Latin square. Fixation displays were presented for 500 ms, after which the search display appeared and remained onscreen for 3,000 ms or until a response was made, whichever occurred first. Visual feedback was given in the form of the appropriate message declaring the response “CORRECT,” “INCORRECT,” or “TOO SLOW.” Manual RTs and accuracy were recorded for each trial.

### Results and discussion

The analyses we report in Experiments 2 and 3 are not identical to those conducted in Experiment 1, reflecting the fact that these experiments were independently conducted by separate groups of researchers. For the following experiments, the analyses strictly followed our preregistration (see the link above). Our analysis procedures were similar to Vatterott and Vecera (2012), except that, due to the online nature of our experiment, we removed participants in two stages. First, we implemented an automatic removal process consisting of two participant exclusion criteria: (1) RTs shorter than 250 ms or no response on 30% or more of all experimental trials and (2) less than 60% accuracy during one or more blocks. Two participants were removed based on the latter criterion and were not included in calculation of averages for our second stage of participant removal. Second, we excluded any participants with a mean RT or accuracy outside of a 2.5 standard deviation range from overall participant averages. Three additional participants were removed for this reason. Data from the remaining 60 participants were included in the final analyses described below. Within each participant, we excluded trials on which RT fell outside the range of 3 standard deviations from the relevant condition mean. Trials on which an error was made (i.e., incorrect or no response) were excluded from RT analyses. These criteria resulted in the exclusion of 6.2% of all trials, which is similar to the value of 7.7% reported for Experiment 1. The Holm–Bonferroni procedure was used to correct for multiple comparisons (Holm, 1979).

#### Response times

##### Split-block analyses

**Fig. 3:**
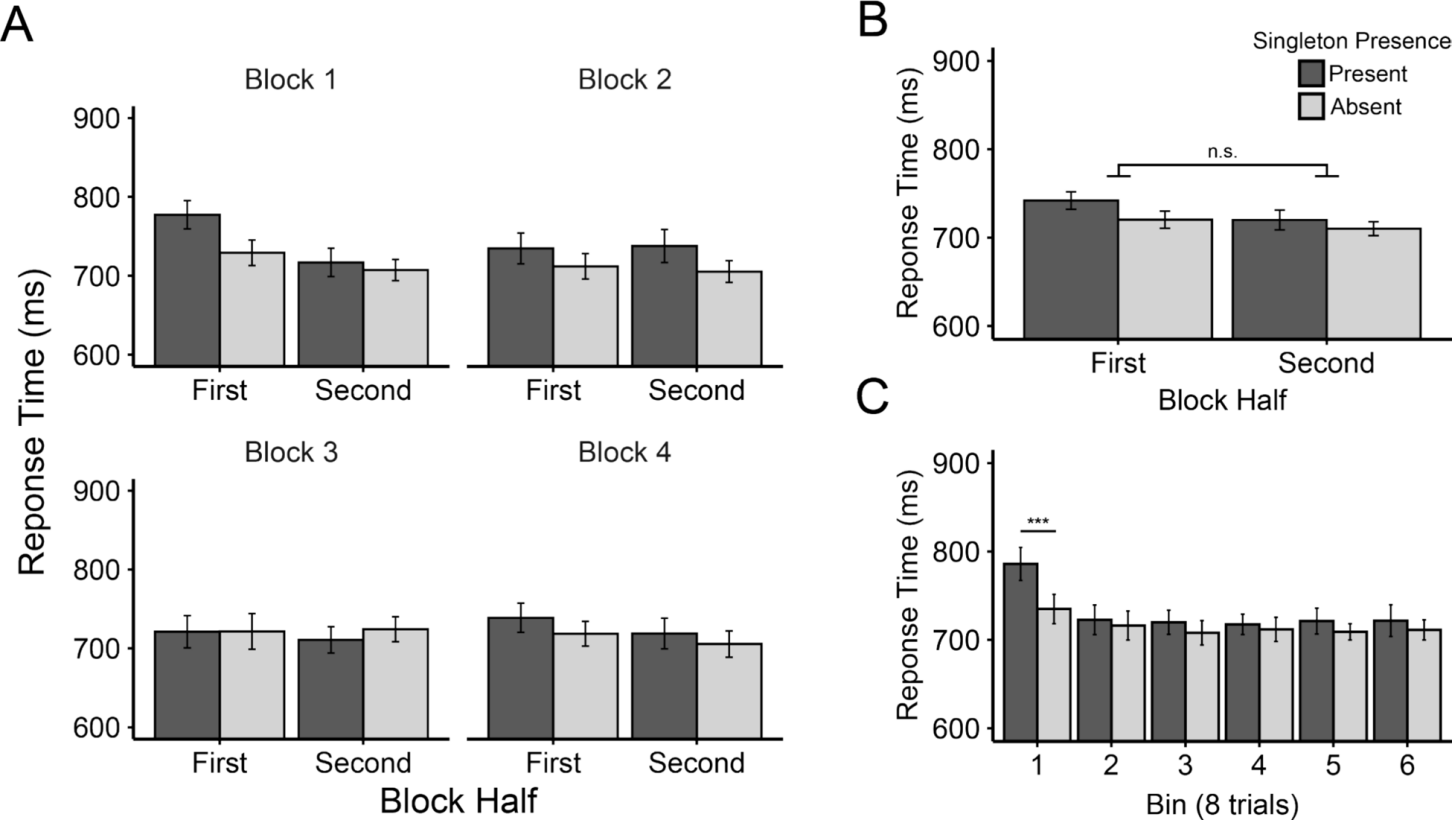
Mean response time (in ms) from Experiment 2 (***A***) displayed as a function of singleton presence, block half, and block. ***B*** Response times presented in the same manner as panel A but pooled across blocks. ***C*** Response time collapsed across blocks as a function of singleton presence across six bins (each containing eight trials). Error bars represent within-subject 95% confidence intervals. *** indicates *p* < .001

Our primary analyses were identical to those conducted by Vatterott and Vecera (2012). We first investigated the attenuation of attentional capture across block halves through a 2 × 2 repeated-measures ANOVA with factors singleton presence (Present vs. Absent) and block half (First vs. Second 24 trials of each block). For this analysis we collapsed across all blocks of the experiment, with the assumption that each block should show a similar pattern of results (Fig. 3B). We observed significant main effects of singleton presence, *F*(1, 59) = 13.82, *p* < .001, *η*_*p*_^*2*^ = .19, and block half, *F*(1, 59) = 6.45, *p* = .014, *η*_*p*_^*2*^ = .10. RTs were slower when the singleton was present (731 ms) than when it was absent (715 ms), indicating attentional capture by the singleton distractor. RTs were also slower for the first halves of blocks (731 ms) than the second halves of blocks (715 ms). The interaction between the two was not significant, *F*(1, 59) = 2.61, *p* = .112, *η*_*p*_^*2*^ = .04, inconsistent with Vatterott and Vecera (2012).

To gauge attention capture in each half of the collapsed blocks we performed two preplanned paired-samples *t* tests, which compared singleton-present and singleton-absent trials in each block half. These tests revealed that, similar to Vatterott and Vecera (2012), singleton-presence costs were significant in the first half of the collapsed blocks (mean diff. = 22 ms), *t*(59) = 3.82, *p* < .001, *d*_z_ = 0.49, but were only marginally so in the second half (mean diff. = 10 ms), *t*(59) = 1.78, *p* = .080, *d*_*z*_ = 0.23.

Our next analysis examined whether the results varied as a function of block. We calculated singleton presence costs as the RT difference between singleton present and absent trials, then entered them into a 2 × 4 repeated-measures ANOVA with block half (first vs. second) and block (1–4) as factors. The main effect of block was significant, *F*(3, 177) = 4.49, *p* = .005, *η*_*p*_^*2*^ = .07, with greater singleton presence costs in Blocks 1 (28 ms) and 2 (27 ms) compared with Blocks 3 (−7 ms) and 4 (16 ms). An inspection of Fig. 3A appears to show a stronger effect of block half in the first block than the latter blocks, but there was no convincing statistical support for this via the Block Half × Block interaction, *F*(2.51, 148.08) = 2.38, *p* = .083, *η*_*p*_^*2*^ = .04. We will return to this issue in the Pooled Data Analysis and General Discussion.

##### Finer-grained analyses (eight-trial bins)

We next conducted an analysis similar to the singleton presence × block half ANOVA above, but on a somewhat more fine-grained time course. Instead of using block halves, we divided each block into six bins, each containing eight trials. We then collapsed across blocks and conducted a 2 × 6 repeated-measures ANOVA with singleton presence (Present vs. Absent) and bin (1–6) as factors (Fig. 3C). There was a significant main effect of bin, *F*(3.13, 184.53) = 10.09, *p* < .001, *η*_*p*_^*2*^ = .15, which appeared to be driven by slower RTs in the first bin (760 ms) compared to all others (max = 720 ms, min = 714 ms). Critically, the interaction of singleton presence and bin was significant, *F*(4.04, 238.09) = 3.43, *p* = .009, *η*_*p*_^*2*^ = .06, with smaller singleton presence costs in the second–sixth bins (max = 12 ms, min = 6 ms) relative to the first (51 ms; see Fig. 3C). This was confirmed by follow-up paired-samples *t* tests comparing present and absent trials in each bin individually. Significant attentional capture was observed only in the first bin, *t*(59) = 4.50, *p*_*HB*_ < .001, *d*_*z*_ = 0.58, all other *t*s ≤ 1.59, *p*_*HB*_s ≥ .583, *d*_*z*_s ≤ 0.21.

In Experiment 2, we again did not observe the learned distractor rejection effect when using Vatterott and Vecera’s (2012) split-block analysis (first 24 trials vs. final 24 trials). However, when dividing blocks into bins of 8 trials, the attenuation of capture across the course of the collapsed blocks became clear. As for our across-block pattern of learned distractor rejection, when dividing by block halves, the initial capture followed by rejection was only apparent in the first block, as was found in Experiment 1. Blocks 2–4 did not show an initial capture effect, leaving no room for subsequent rejection. There are two likely explanations for this pattern: (1) the RT cost of capture was greatly reduced in Blocks 2–4 compared with the first block (see Müller et al., 2009; Zehetleitner et al., 2012) or (2) capture occurred on fewer of the early trials in Blocks 2–4 because distractor rejection was learned much more quickly (i.e., fewer encounters with the distractor were required to reject it). It is further possible that some combination of these two options occurred.

#### Error rates

All analyses conducted on RTs, as described above, were additionally conducted on error rates. No significant effects were found, *F*s ≤ 2.44, *p*s ≥ .124, *η*_*p*_^*2*^ ≤ .04.

## Experiment 3

Because Vatterott and Vecera’s (2012) original experiment was conducted in a laboratory setting rather than online, it is conceivable that the deviations from their results observed in the present Experiment 2 were due to the reduced experimental control inherent in online experimentation. The purpose of Experiment 3 was to verify the findings of Experiment 2 in an in-lab setting.

### Method

All experimental procedures and statistical analyses of Experiment 3 were preregistered (10.17605/OSF.IO/QWUFT).

#### Participants

Sixty-two participants (42 women, 18 men, mean age = 18.7 years) were recruited from undergraduate psychology courses at The Ohio State University. This sample size matched that of Experiment 2. Participants were awarded course credit for their participation, which lasted approximately 15 min on average. All participants reported normal or corrected-to-normal color vision and visual acuity. All experimental procedures were approved by The Ohio State University Institutional Review Board. Data were collected in the fall of 2023.

#### Apparatus and stimuli

Experimental stimuli were displayed on a 24-in. Dell G2422HS monitor, with a 1,920 × 1,080 resolution. Participants sat in a dimly lit room approximately 70 cm from the display; head position was not fixed. Stimuli were coded and presented using PsychToolbox3 (Brainard, 1997; Kleiner et al., 2007; Pelli, 1997). Stimuli were identical to those used in Experiment 2, except as noted below.

Apparent shape size (visual angle) differed slightly from Experiments 1 and 2. The target circle diameter measured 2.27°, distractor diamonds and squares were 1.94° on a side and triangle base and height were 2.27°. All shapes were presented at an eccentricity of 4.1° visual angle. All other visual angle measurements differed by less than 0.1° from those reported in Experiment 2.

#### Design and procedure

In order to more closely replicate Vatterott and Vecera (2012), a few minor alterations were made from the procedure used in Experiment 2. Prior to search onset the fixation display was presented for 1,000 ms, then participants were allowed 5,000 ms to report detection of the target while the search array remained on screen. Visual feedback was presented only when an error was made or when no response was given within the allotted time.

### Results and discussion

Participant removal procedures were identical to Experiment 2. One participant was removed due to the criterion requiring an accuracy greater than 60% in all experimental blocks. One additional participant was excluded from analyses because their accuracy fell outside of the 2.5 standard deviation range from the mean of all participants. We retained these relatively strict removal criteria to remain consistent with Experiment 2, however the inclusion of the first removal stage had no effect on our reported pattern of results. Individual trial removal resulted in a removal rate of 5.6%.

#### Response times

##### Split-block analyses

**Fig. 4:**
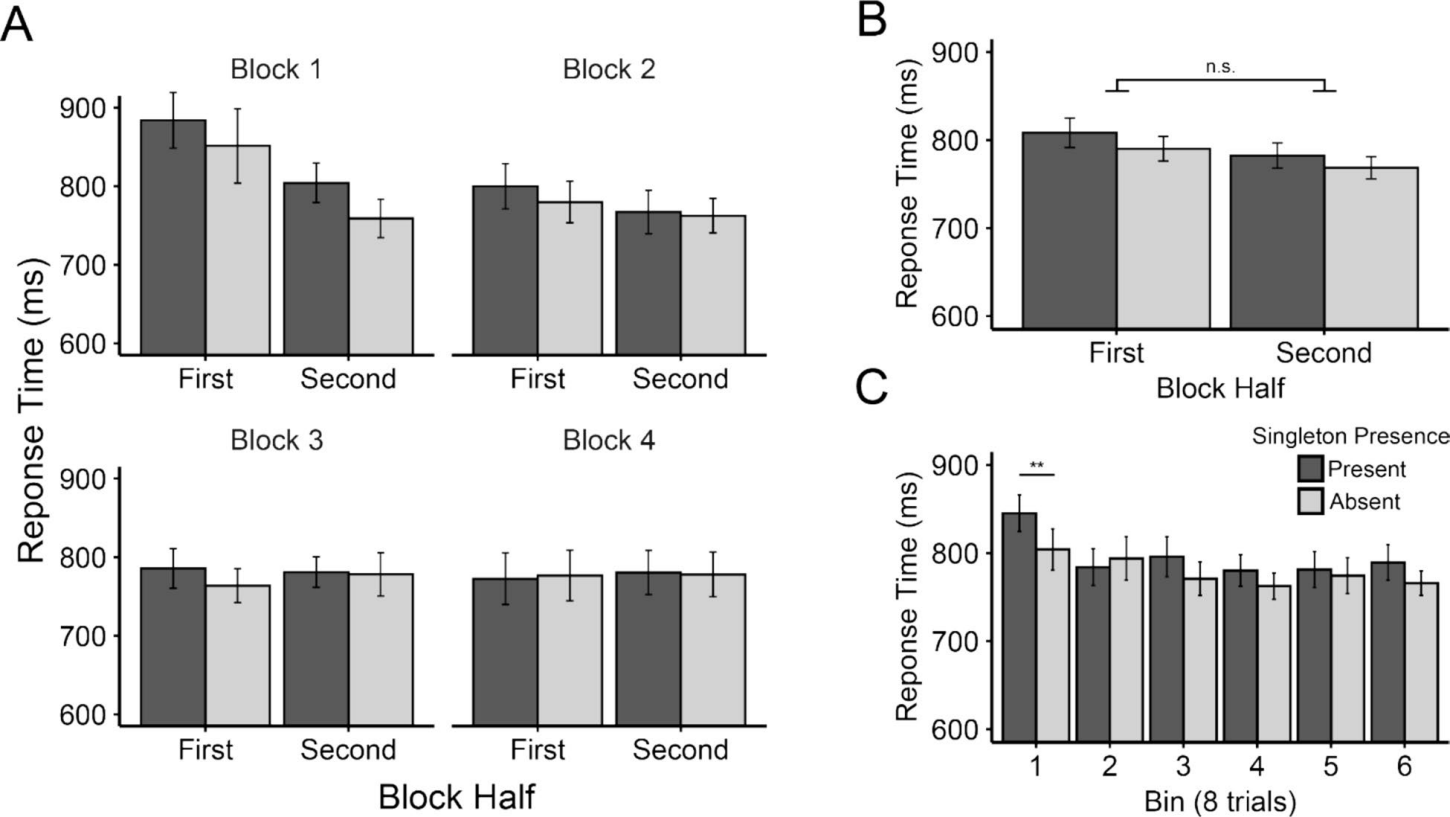
Mean response times (in ms) from Experiment 3 (***A***) displayed as a function of singleton presence, block half, and block. ***B*** Response time presented in the same manner as panel A but pooled across blocks. ***C*** Response time collapsed across blocks as a function of singleton presence across six trials bins (each containing eight trials). Error bars represent within-subject 95% confidence intervals. ** indicates *p* < .01

Our main analyses were the same as in Experiment 2. The singleton presence by block half ANOVA, conducted across collapsed blocks, produced the same pattern of results observed in Experiment 2 (Fig. 4B). The main effect of singleton presence was significant, *F*(1, 59) = 5.28, *p* = .025, *η*_*p*_^*2*^ = .08, with slower RTs on singleton present trials (795 ms) than singleton absent trials (779 ms). In addition, a significant main effect of block half was found, *F*(1, 59) = 6.69, *p* = .012, *η*_*p*_^*2*^ = .10. RTs were slower in the first half (799 ms) relative to the second half (776 ms) of collapsed blocks. More critically, however, the two did not interact significantly, *F*(1, 59) = 0.21, *p* = .652, *η*_*p*_^*2*^ = .003.

Paired-samples *t* tests surprisingly revealed marginal capture effects in both the first, *t*(59) = 1.87, *p* = .066, *d*_*z*_ = 0.24, and the second, *t*(59) = 1.91, *p* = .061, *d*_*z*_ = 0.25, halves of the collapsed blocks (18 ms and 14 ms, respectively; see Fig. 4B). Our analysis of smaller bins of trials below sheds some light on this potentially puzzling result.

To determine whether the results varied across blocks, we examined singleton presence costs as a function of block half and block in a 2 × 4 repeated-measures ANOVA. Our results were similar to Experiment 2, with a significant main effect of block, *F*(2.566, 151.372) = 3.05, *p* = .038, *η*_*p*_^*2*^ = .05, reflecting a decline in singleton presence costs across blocks. However, we did not find a statistically significant interaction between block half and block, *F*(3, 177) = 0.597, *p* = .618, *η*_*p*_^*2*^ = .01 (see Fig. 4A).

##### Finer-grained analyses (eight-trial bins)

Again, we found the same pattern of results when conducting a finer-grained analysis of attentional capture across the course of collapsed blocks. A 2 × 6 ANOVA comparing singleton presence as a function of eight-trial bins was conducted (see Experiment 2 for description). We found a significant main effect of bin, *F*(5, 295) = 5.89, *p* < .001, *η*_*p*_^*2*^ = .09, and a significant interaction between bin and singleton presence, *F*(5, 295) = 2.25, *p* = .0497, *η*_*p*_^*2*^ = .04 (Fig. 4C). As expected, RTs appeared to be slowest in earlier bins and fastest in later bins but, similar to Experiment 2, the largest difference between two successive bins was between Bins 1 and 2 (827 ms and 789 ms, respectively). RTs across the remaining bins were relatively consistent (min = 771 ms, max = 781 ms). Singleton presence costs were larger in the first bin (43 ms) than the following bins, which showed variable RT costs in no discernable pattern (max = 25 ms, min = −10 ms). Paired-sample *t* tests revealed that singleton presence costs were significant only in the first bin, *t*(59) = 3.35, *p*_*HB*_ = .008, *d*_*z*_ = 0.43, all other *t*s ≤ 2.26 , *p*_*HB*_s ≥ .136, *d*_*z*_s ≤ .29. However, the cost was also significant in Bin 6 before correcting for multiple comparisons (*p*_*raw*_ = .027).

Our results here echo those of Experiment 2. We were unable to detect the learned distractor rejection effect with the conventional split block analysis, but when using bins containing fewer trials the effect became clear. As in our previous experiment, the numerical pattern of capture across blocks was inconsistent with Vatterott and Vecera’s (2012) findings. Numerically, attentional capture appeared primarily in the first block, making it difficult to observe a learned distractor rejection effect in subsequent blocks. There may have been a capture-rebound effect (i.e., an increase in initial attentional capture) in those blocks, but it was simply too short-lived to detect with on a coarse time-scale analysis (i.e., entire block halves). We investigate this further in a combined experiment analysis described below.

#### Error rates

The same analyses described above were conducted on error rates while simplifying the 2 × 2 and 2 × 4 ANOVAs into a single 2 × 2 × 4 ANOVA. This analysis revealed that block half significantly interacted with both singleton presence, *F*(1, 59) = 6.56, *p* = .013, *η*_*p*_^*2*^ = .10, and block, *F*(3, 177) = 4.28, *p* = .006, *η*_*p*_^*2*^ = .07; all other *F*s ≤ 1.17, *p*s ≥ .323, *η*_*p*_^*2*^ ≤ .02. In both cases, error rates followed the same overall pattern as RTs, therefore, no speed–accuracy trade-off was observed. Interestingly, we observed a “learned distractor rejection effect” in error rates across the collapsed blocks with a decrease in error rate singleton presence costs (present–absent) from the first (0.79%) to second (−0.88%) half of the pooled blocks.

No significant effects were found when comparing singleton presence as a function of bin (2 × 6 ANOVA), *F*s ≤ 1.86, *p*s ≥ .101, *η*_*p*_^*2*^ ≤ .03.

#### Comparison of Experiments 2 and 3

Finally, we conducted a comparison of the split block analysis across Experiments 2 and 3 (online and in-person settings, respectively). This analysis was identical to the singleton presence by block half ANOVA described above with the addition of a third factor of experimental setting (online vs. in-lab; a between-subjects variable), resulting in a 2 × 2 × 2 mixed-measures ANOVA. We conducted this analysis on RTs and error rates using both frequentist and Bayesian statistical frameworks. Bayesian analyses followed the procedure outlined in van den Bergh et al. (2020) and used an uninformed prior. Note that we report only the novel comparisons involving the effect of experiment.

In an analysis of RTs across the experiments, We found a significant main effect of experiment, *F*(1, 118) = 7.11, *p* = .009, *η*_*p*_^*2*^ = .06, *BF*_*incl*_ = 3.72, with longer RTs for participants completing the experiment in the lab (787 ms) than online (723 ms). More importantly, however, there were no significant interactions between the factors, *F*s ≤ 0.48, *p*s ≥ .491, *η*_*p*_^*2*^ ≤ .004, *BF*_*incl*_ ≤ 0.27. Furthermore, all interactions involving the experiment factor produced Bayes factors favoring the null hypothesis. Most critically, the singleton presence by block half interaction (split-block analysis) did not differ across experimental settings, *BF*_*incl*_ = 0.27. Additionally, the Block Half × Experiment, *BF*_*incl*_ = 0.27, and Singleton Presence × Experiment, *BF*_*incl*_ = 0.17, interactions produced null-supporting Bayes factors. Therefore, with the exception of overall RTs, we present evidence that RT effects did not differ across the experimental settings.

We also analyzed error rates in the same way. No significant results were found, *F*s ≤ 2. 36, *p*s ≥ .127, *η*_*p*_^*2*^ ≤ .02, *BF*_*incl*_ ≤ 0.53. With respect to Bayes factors, we found only anecdotal support for the null hypothesis for the main effect of experiment, *BF*_*incl*_ = 0.53. As with our RT analysis above, all three interaction effects involving experiment showed more conclusive support for the null, *BF*_*incl*_ ≤ 0.24.

## Pooled data analysis

When Vatterott and Vecera (2012) changed the color of a singleton distractor from block to block, large capture costs (~77 ms) emerged in the first half of that block, then disappeared or reversed in the second half. The absence of this dramatic pattern in the present three experiments seems to be driven by two factors. First, we found that the attenuation of capture at the start of each block happened remarkably quickly, perhaps in as few as two to three distractor encounters. When averaging across an entire block half (i.e., 12 distractor encounters), the attenuation effect washed out. Second, the learned distractor rejection effect was more pronounced in the first block than in subsequent blocks, which showed a lack of robust attentional capture in both block halves. So, the effect likely also averaged out when collapsing across blocks.

The consistency of the learned distractor rejection effect across blocks was critical to Vatterott and Vecera’s (2012) conclusion that learned distractor rejection is primarily feature-based. However, all three experiments reported in the present study failed to find the same consistency. To shed further light on this important data pattern, we applied our fine-grained (trial by trial) analysis to individual blocks. To obtain sufficient power, we carried out exploratory analyses of the combined data from all three experiments, giving us a pooled sample of 190 participants.

### Split-block analyses

**Fig. 5:**
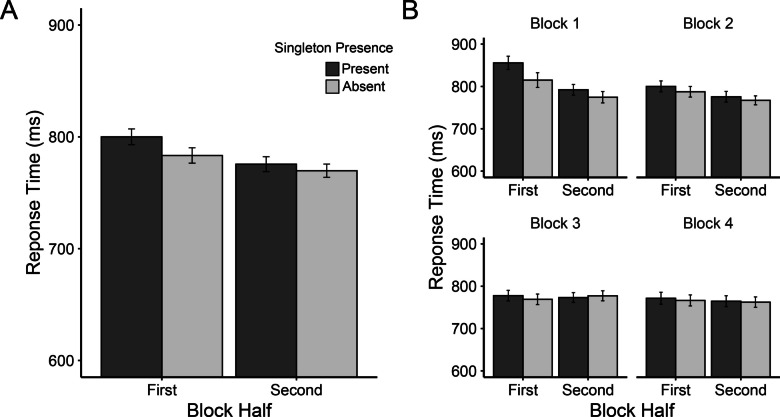
Mean response time (in ms) from the pooled samples of Experiments 1–3 as a function of singleton presence and block half (***A***) collapsed across blocks and (***B***) in each individual block. Error bars represent within-subject 95% confidence intervals

We first examined the numerical pattern of singleton presence costs across block halves (Fig. 5A) and across blocks (Fig. 5B). As expected, the pooled data pattern is very similar to what we reported in Experiments 1–3. Specifically, in Block 1, attentional capture in the first half of the block was quite large (39 ms), with a substantial decrease in capture in the second half of the block (18 ms). This pattern was less apparent in the latter blocks (2–4), where we see only minimal attentional capture in the first halves of blocks overall (12 ms, 11 ms, and 5 ms for Blocks 2–4, respectively), leaving little room for further reduction by the second block half (8 ms, −4 ms, and 3 ms).

### Moving RT analyses

**Fig. 6:**
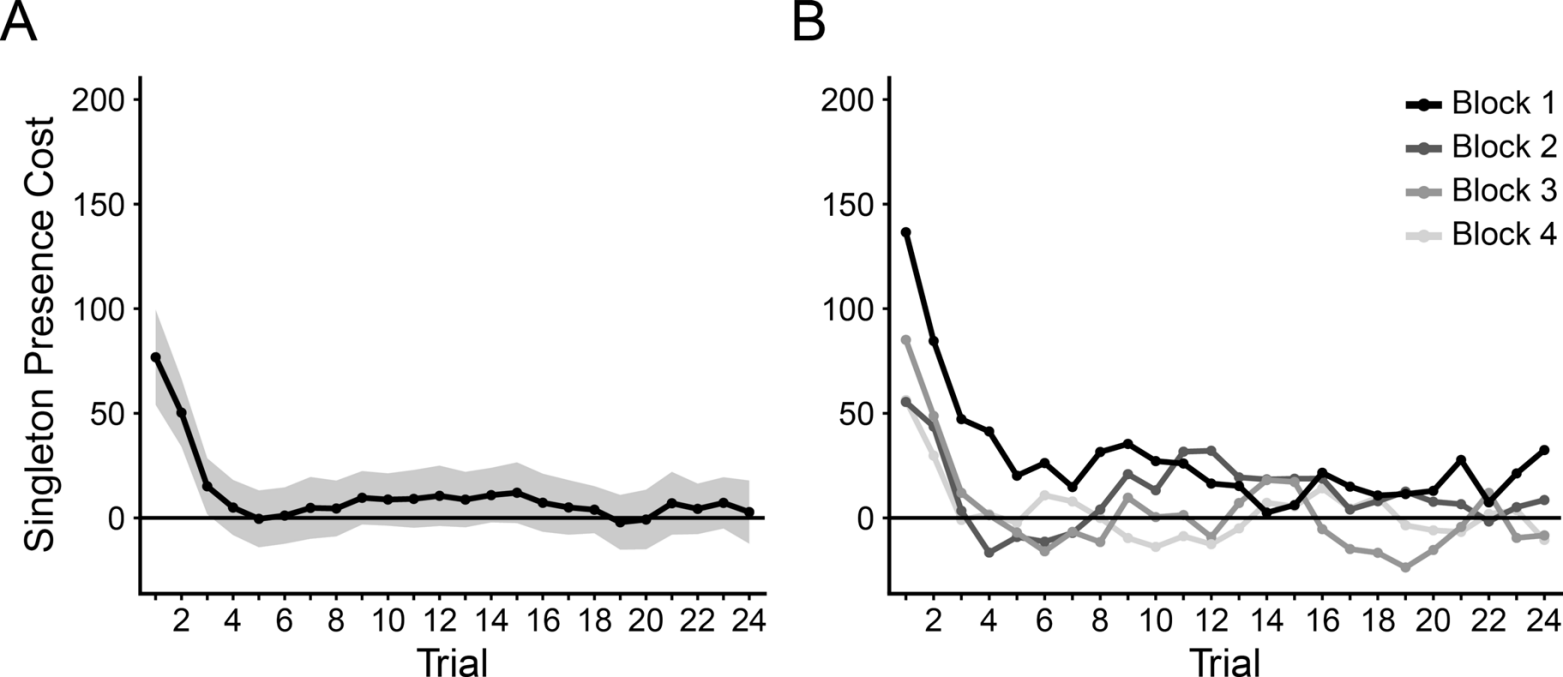
Moving average of singleton presence costs from the combined samples of Experiments 1–3. Each “trial” represents the average of itself and the two neighboring trials of the same condition (Present vs. Absent). Singleton presence costs were calculated as the Present-Absent RT difference of “trials” generated in this way. ***A*** Singleton presence costs collapsed across all four blocks of the experiment. The ribbon represents a within-subject 95% confidence interval at each trial. ***B*** Singleton presence costs in each block individually

To analyze the pooled data from all three experiments on a finer-grained time scale, we focused on the moving RT analysis described in Experiment 1, except that we used a three-trial window instead of a five-trial window. Each data point consisted of the average of only 12 (or three in the case of the block-by-block analysis) measurements per participant for each condition but was feasible due to the size of our combined sample size (*N* = 190). Singleton presence costs for each trial collapsed across blocks are depicted in Fig. 6A. To determine whether a significant capture effect occurred for each individual trial, we conducted a Wilcoxon signed rank test comparing RTs on present and absent trials.^3^ We chose a nonparametric approach due to the nonnormality of the RT distributions on individual trials.

A significant difference between RTs on singleton present and absent trials was found only on Trial 1 (Mean diff. = 77 ms, *Z* = 5.70, *p*_*HB*_ < .001) and Trial 2 (Mean diff. = 50 ms, *Z* = 5.63, *p*_*HB*_ < .001), with all other Mean diffs. ≤ 15 ms, *Z*s ≤ 2.00, *p*_*HB*_s = 1.00. Correcting for 24 comparisons using the Holm–Bonferroni procedure did not change the significance of any of our comparisons (but is the reason that the corrected *p* values on Trials 3–24 are equal to 1.00). Although we lack clear statistical support for capture beyond the second trial, an inspection of Fig. 6A shows that the pattern continues to decline numerically from Trial 3 to Trial 4 (the uncorrected *p* value on Trial 3 was *p* = .051).

We also took advantage of the large number of participants in the combined sample to investigate the block-by-block pattern of RTs using the moving RT analysis. As shown in Fig. 6B, the initial capture by the color singleton, followed by a rapid rejection, occurred in each block individually, supporting Vatterott and Vecera’s (2012) claim of feature-dependent distractor rejection. However, it is also apparent (numerically) that the magnitude of the early-trial capture effect decreased across the blocks. Numerically, the largest capture effect was observed in Block 1 (average across first three trials = 87 ms), with substantially less capture occurring at the start of Blocks 2–4 (34 ms, 53 ms, and 37 ms, respectively). To formally analyze this pattern, singleton presence costs averaged across the first three trials of each block were entered into a one-way repeated-measures ANOVA, with block (1–4) as the only factor. We additionally tested for a negative linear trend across blocks. Results showed a marginal effect of block on singleton presence costs, *F*(2.85, 532.17) = 2.26, *p* = .084, *η*_*p*_^*2*^ = .01, and a marginal linear trend, *F*(1, 561) = 3.68, *p* = .055, *η*_*p*_^*2*^ = .007. Though we did not find convincing statistical evidence for a decrease in capture across blocks using the analyses above, it could be argued that a more sensitive test of this pattern would be to compare Block 1 to the average of Blocks 2–4 (i.e., a contrast of [3, −1, −1, −1] for the four respective blocks). Such a test relies heavily on post-hoc speculation, so we offer the result but caution against overinterpretation, *F*(1, 561) = 6.33, *p* = .012, *η*_*p*_^*2*^ = .01. Future studies are necessary to verify this effect.

## General discussion

Here, we described three attempted replications of Vatterott and Vecera (2012) that were independently conducted by two groups of researchers using three different participant pools (online participants from Prolific.co as well as undergraduates from both Oregon State University and The Ohio State University). When directly replicating the design and analyses of the original study as closely as possible, we were unable to replicate the dramatic pattern of results reported by Vatterott and Vecera. Specifically, when comparing singleton presence costs between the first and second halves of collapsed blocks, we did not observe a large and significant attenuation of attention capture costs.^4^ Thus, at first glance, we seemed to fail to replicate their findings.

However, upon further analysis, it became evident that our “failure to replicate” was not quite what it initially seemed. Finer-grained analyses using either the moving RT approach (with a window of three or five trials) or bins of fewer trials (eight rather than 24) revealed a substantial capture effect in the beginning of blocks that completely attenuated after only a few encounters with the new distractor color. The bin analysis revealed significant singleton presence costs only in the first bin of eight trials, suggesting that the distractor was efficiently rejected in less than four encounters. The moving average analysis clarified that only two to three encounters with the distractor were required for the elimination of capture costs. Together, these results demonstrate that the relatively coarse 24-trial split-block analysis used by Vatterott and Vecera (2012) obscures the true duration of the early-trial capture effect—and will often be insufficient to even detect its presence. Notably, Vatterott and Vecera acknowledged that their analysis may have been too coarse, and reported that a visual inspection of their data on a trial-by-trial basis showed that capture disappeared after only eight or nine encounters with the salient distractor.

Gaspelin and Luck (2018), who inspired our moving RT analysis, also found a short-lived capture effect that quickly attenuated. In their Experiment 4, they analyzed first saccades on each trial, using a moving window of 11 trials (±5 trials). They found that participants were more likely to initially fixate the color singleton distractor compared to target-colored distractors (oculomotor capture), for the first several trials upon encountering a new distractor color. Though Gaspelin and Luck did not report the timepoint at which the oculomotor capture effect was no longer significant, it seemed to take approximately eight to 12 trials (i.e., ~4–6 distractor present trials) based on an inspection of their Fig. 8. In addition to the current experiments, Gaspelin and Luck’s findings demonstrate that learned distractor rejection likely occurs much faster than the conventional split-block analysis would suggest.

### Recommendations for future research

**Fig. 7:**
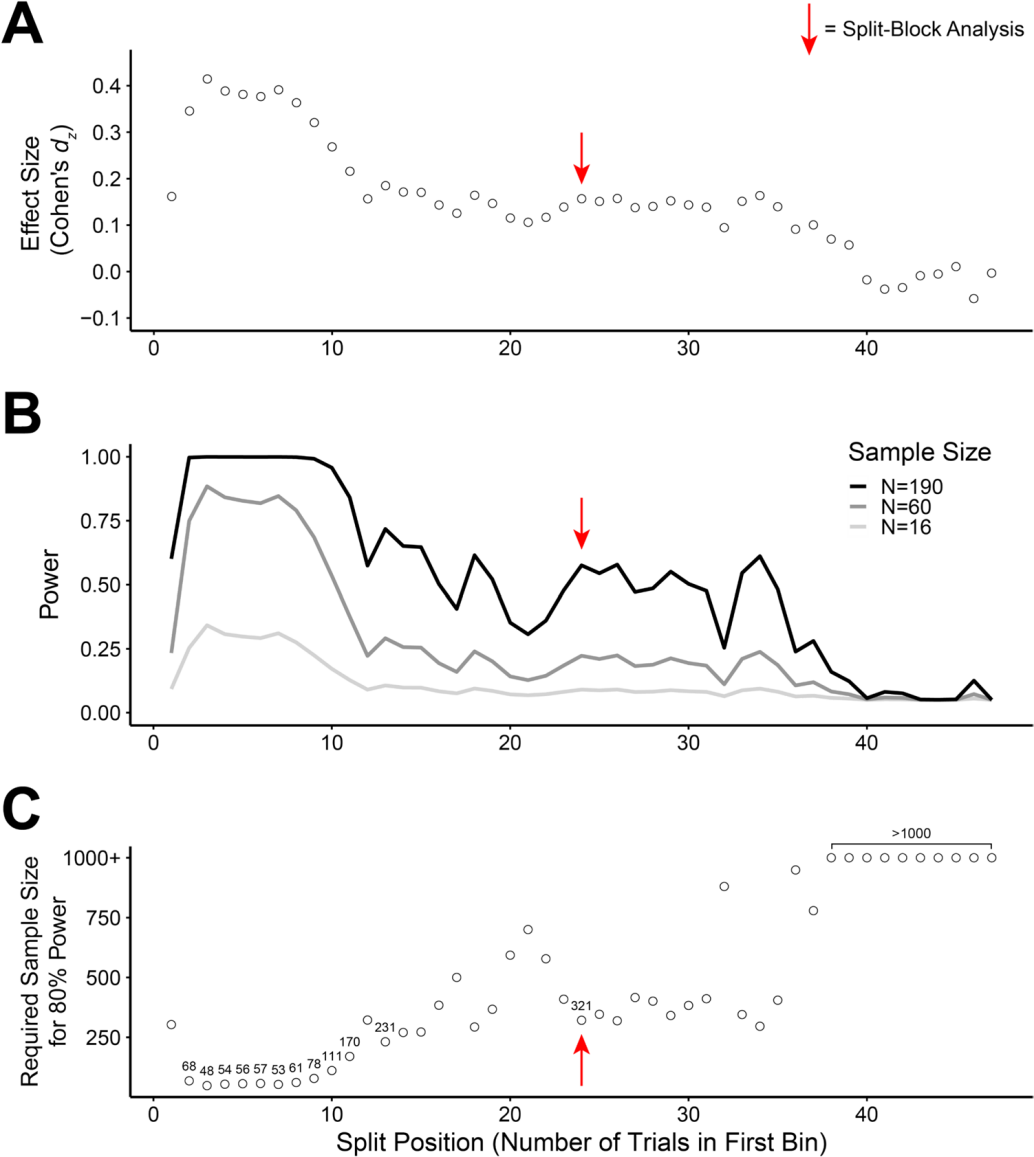
An exploratory investigation of how best to split trials to detect a learned distractor rejection effect. ***A*** Effect size (Cohen’s d_z_) at every possible split of trials into two bins (early trials vs. late trials). ***B*** Obtained (post hoc) power across the trial splits as a function of sample size. Effect sizes for this power analysis were estimated from the pooled sample across Experiments 1–3 (*N* = 190), shown in Panel A. ***C*** Sample size required for 80% power to detect the learned distractor rejection effect (a difference in singleton presence costs across the split) across all trial splits. All sample size values less than 250 and the conventional split-block analysis are labeled. Each trial split with 38 or more trials in the first bin yielded a required sample size greater than 1,000 participants

Given that the effects of learned distractor rejection are so short-lived and thus difficult to expose with small sample sizes, how might researchers maximize their chances of reliably measuring this phenomenon? To address this question, we leveraged our pooled sample to estimate how to obtain the most reliable and power-efficient characterization of the learned distractor rejection effect. We applied the logic of the split-block analysis, which usually compares the first 24 trials to the second 24 trials of the block, to every possible binary split of trials (i.e., Trial 1 vs. Trials 2–48, Trials 1–2 vs. Trials 3–48, etc.). After collapsing across blocks, we conducted 47 paired-samples *t* tests on singleton presence costs across every such *trial split*. After correcting for multiple comparisons, the *t* tests with the first bin containing anywhere from two to ten trials were significant, *t*s ≥ 3.70, *p*_*HB*_s ≤ .011, *d*_*z*_s ≥ 0.27. No other tests produced significant results, *t*s ≤ 2.98, *p*_*HB*_s ≥ .126, *d*_*z*_s ≤ 0.22. However, future studies are unlikely to conduct tests at every possible split, as we do here. Therefore, we would also like to note that splits with the following number of trials in the first bin produced significant results before correcting for multiple comparisons: 11–16 trials, 18–19 trials, 24–26 trials, 29–30 trials, and 33–34 trials, *t*s ≥ 1.98, *p*_*raw*_ ≤ .0496, *d*_*z*_s ≥ 0.14. Naturally, effect sizes followed the same pattern with the largest effect size in the 3:45 comparison (*d*_*z*_ = 0.41). The other comparisons between 2:46 and 10:38 produced similar effect sizes (min *d*_*z*_ = 0.27, max *d*_*z*_ = 0.39). Comparatively, the conventional split-block analysis (24:24) produced an effect size of only *d*_*z*_ = 0.16. Estimated Cohen’s *d*_*z*_s across all splits are shown in Fig. 7A.

One might next question how much statistical power can be obtained across the different splits, using various sample sizes. To address this, we used the effect sizes obtained above to calculate the achieved power across all of the splits, using sample sizes of *N* = 190, 60, and 16. Finally, we determined the sample size required at each split to obtain significant results with 80% power (using the *pwr* package in R; Champely, 2006). Results are depicted in Fig. 7B–C, respectively. For the conventional split block analysis (24:24), even with our large sample of 190 participants we obtained power of only .58. Our sample size of 60 from Experiments 2 and 3 only provided power of .22. Meanwhile, Vatterott and Vecera’s (2012) sample size of 16 participants would have resulted in power of .09. Perhaps most strikingly, an a priori power analysis based on the effect size of the 24:24 split block analysis from the full sample of *N* = 190 revealed that future experiments using this split would need to recruit a sample of 321 participants to achieve 80% power. However, choosing a split with between two and ten trials in the first bin has much better power prospects. For example, comparing the first six trials of the block to the remaining 42 would require a sample of only 57 participants to obtain 80% power. All things considered, we recommend the retirement of the 24:24 split-block analysis. Instead, finer grained analyses (e.g., the moving RT or binned analyses) or a more sensitive split, such as the options outlined above, should be used.

#### Learned distractor rejection across blocks

In addition to the speed at which rejection was learned, we identified a second potential factor contributing to the lack of the canonical split-block learned distractor rejection effect. That is, the numerical effect was not consistent across experimental blocks. Although the first block produced an obvious effect across block halves, the proceeding blocks did not. The purpose of Vatterott and Vecera’s (2012) manipulation of the distractor color was to test the feature specificity of learned distractor rejection through repeatedly eliciting the early-trial capture effect. Importantly, this makes the consistency of the learned distractor rejection effect across blocks critical to their proposal of a feature-dependent rejection mechanism.

A finer grained analysis was able to shed some light on this pattern of results. It is clear from our combined analyses that an early trial capture effect did occur in Blocks 2–4, indicating a role for feature-specific rejection as suggested by Vatterott and Vecera (2012) and others (Gaspelin & Luck, 2018; Savelson & Leber, 2024; Stilwell et al., 2019). However, the magnitude of this effect appeared numerically (though not significantly) larger in the first block of the experiment compared to the latter blocks (87 ms vs. 34 ms, 53 ms, and 37 ms, in Blocks 1–4, respectively). There are a number of proposed mechanisms within the attentional capture and distractor rejection literature that could account for this apparent feature-independent decrease in the early-trial capture effect across blocks. However, in light of the lack of statistical evidence for this effect we will limit or speculation on this pattern (but see Ma & Abrams, 2023; Vatterott et al., 2018; Won et al., 2019, for recent work on feature-independent distractor ignoring).

Previous work has shown that the attentional capture associated with a salient distractor is much greater the first few times it is encountered (Adam et al., 2024; Ernst et al., 2020; Horstmann, 2002; Müller et al., 2009; Zehetleitner et al., 2012; but see Gibson & Jiang, 1998). Note that here we refer to the first time encountering *any* salient distractor, not different distractors. That is, if participants are not used to seeing salient distractors in the search array *at all,* then the initial capture by the first distractor they see will be much greater. Later, when a novel distractor is presented, it may not produce the same magnitude of attention capture simply because participants are used to seeing distractors in the display.

Importantly, regardless of the possibility of reduced capture in Blocks 2–4, our pattern of results is consistent with feature-specific distractor rejection. Capture occurred on the first few trials of each block, but quickly attenuated as rejection of the novel color was learned. The evidence we present here does not disagree with Vatterott and Vecera’s (2012) original formulation of learned distractor rejection. However, we show that the learning process occurs more rapidly than they assumed, and that the rejection of distractors may be more robust to changes in basic features than was apparent in the original study.

#### Relation to previous studies

Again, we would like to point out that though we are the first to directly replicate Vatterott and Vecera’s (2012) methodology and analyses, others have attempted conceptual replications. Notably, some of these conceptual replications were successful in producing a statistically significant (but smaller) split-block learned distractor rejection effect (De Tommaso & Turatto, 2019; Ramgir & Lamy, 2023; Vatterott et al., 2018), while others encountered significant difficulties (Gaspelin & Luck, 2018; Savelson & Leber, 2024).

While we cannot be certain, we speculate that the discrepancy in results may be due to between-sample variability in the magnitude (but not duration) of the early-trial capture effect. That is, in each of these conceptual replications (and in Vatterott & Vecera’s, 2012, original study), the capture effect is likely very short lived, similar to what we find in our present experiments. Indeed, we conducted a trial-by-trial visual inspection of Savelson and Leber’s (2024) Experiment 2 which confirmed this to some extent. Despite obtaining a significant split-block learned distractor rejection effect, the initial capture effect in the Savelson and Leber’s study had a similar duration to what we report here, lasting only two to three encounters with the distractor (see also Gaspelin et al., 2019; Gaspelin & Luck, 2018). Thus, what determines a significant block-half effect may be whether the magnitude of the effect in the first few trials is great enough to pull up the average capture score across the first block half as a whole. This could occur with a large effect in studies using relatively small sample sizes (e.g., *N* = 20 in De Tommaso & Turatto, 2019; 16 in Vatterott & Vecera, 2012) or with smaller effects in studies with large sample sizes (e.g., *N* = 96 in Ramgir & Lamy, 2023; 300 in Savelson & Leber, 2024). Regardless of the true reason for the discrepant findings in the split-block analyses, the present experiments demonstrate the necessity of using a finer-grained approach to analyzing the learned distractor rejection effect in order to properly understand its true nature.

In addition to the attempted replications of Vatterott and Vecera’s (2012) work discussed earlier, many other studies have investigated the feature-based learned ignoring of irrelevant stimuli (Stilwell & Vecera, 2019, 2022a, b; Won & Geng, 2018). However, in these studies, the critical “distractor” search items were often nonsalient and therefore would not be expected to (and did not appear to) capture attention in the same way a salient color singleton would. Despite this, these studies provide potentially critical insights into the current results, and our understanding of learned distractor rejection as a whole. Won and Geng (2018), for instance, tested the “spreading” of feature-based distractor rejection to items containing similar features. They found that distractor items were rejected even if they appeared in a color that was merely similar to a previously encountered distractor. In the current experiments (and in Vatterott & Vecera, 2012), the four colors we used (red, yellow, purple, orange) might have been considered “similar” in the context of Won and Geng’s (2018) experiment. Therefore, it is possible that the feature-dependent effect we were trying to observe would have benefited from choosing more distant colors to serve as our salient distractors. However, it is possible that the salience of the distractors we used could override the broad tuning of distractor rejection that Won and Geng found given that attending to salient items (that haven’t been deemed irrelevant) likely serves an important function in the world at large.

We would also like to note that while we have interpreted learned distractor rejection effects as feature-specific in nature, we acknowledge the possibility that the phenomenon is instead due (at least in part) to the dissipation of surprise associated with encountering a new distractor color (Ernst & Horstmann, 2018; Horstmann, 2002, 2015a, b; see also Ernst et al., 2020). Disentangling the relative roles of feature-specific rejection and surprise is a challenging proposition and beyond the scope of the present work but is an important topic for future research.

One final consideration is that the present task encouraged a feature search mode for a specific target shape (Bacon & Egeth, 1994). It remains to be seen whether the time course of learned distractor rejection would be similar under other search strategies, such as singleton detection mode, which is associated with greater—and more persistent—distraction effects (see De Tommaso & Turatto, 2019).

## Conclusions

To summarize, we provide evidence necessitating an update to how learned ignoring of distractors is studied in the field as a whole. The split block analysis may be sufficient to detect the learned distractor rejection effect in some cases (De Tommaso & Turatto, 2019; Ramgir & Lamy, 2023; Vatterott et al., 2018), but not others (Gaspelin & Luck, 2018; Savelson & Leber, 2024). In the present work, we show that analyzing learned distractor rejection in this way is unreliable at best, requiring an estimated sample size of 321 participants to achieve 80% power. Further, it offers an insufficient characterization of the processes of learning to ignore distractors. The rapidity with which distractors can be learned to be rejected is theoretically relevant to some of the most prominent accounts of distractor ignoring—namely, the signal suppression hypothesis (Gaspelin et al., 2015; see also Gaspelin & Luck, 2019), the habituation account of attentional capture (De Tommaso & Turatto, 2019; Turatto & Pascucci, 2016; Won & Geng, 2020; see also Turatto, 2023), and accounts of statistically learned distractor suppression (Stilwell et al., 2019; Stilwell & Vecera, 2019; Wang & Theeuwes, 2018a, b). Likewise, these accounts tend to place heavy emphasis on the necessity of experience with consistent distracting items for their proposed mechanisms to operate. Taking a finer-grained approach to analyzing the time course of learning to reject, or suppress, distractors could provide important—and often theoretically overlooked—insight into the mechanisms underlying distractor ignoring.

## Data Availability

All data from the current experiments are available online at https://osf.io/yxg8e (Experiment 1), https://osf.io/ybdq7/ (Experiments 2 and 3).

## Footnotes

1 Vatterott and Vecera (2012) did not report the effect size with their sample size of 16. In addition, they did not report any participant exclusion criteria.

2 Here, and in the following experiments, demographic information is reported for participants retained in the final analyses. See results section for participant removal procedures. Also, note that participants were only given the choice between “Male” and “Female” when selecting their gender identity in Experiment 1. Therefore, the gender distribution we report here may not reflect the gender identity of the participating individuals.

3 Not every participant had an average RT for both present and absent trials at each data point due to the randomization of present and absent trials during the experiment in combination with trial removal procedures. Participants were excluded from a given *t* test if they were missing one or both RT values required for that test (which occurred only for Trials 1 and 20).

4 We did, however, observe this effect in error rates in Experiment 3, likely as the result of some sort of speed–accuracy trade-off in this effect. Therefore, we do see some evidence for the canonical learned distractor rejection effect in one of our experiments, though in a different form than expected.

## Code availability

Data analysis scripts for Experiments 2 and 3 are available online at https://osf.io/ybdq7/
